# Comparative Evaluation of Surface Roughness, Wettability, and Hardness of Conventional, Heat-Polymerized, Computer-Aided Designed and Milled, and Three-Dimensionally Printed Polymethyl Methacrylate Denture Base Resins: An In Vitro Study

**DOI:** 10.7759/cureus.85008

**Published:** 2025-05-28

**Authors:** Balbir Singh, Shashikala Jain, Navreet Bhasin, Jasbir Kaur, Priyanka Borse, Pursenla Longkumer

**Affiliations:** 1 Department of Prosthodontics, Maharaja Ganga Singh Dental College and Research Centre, Sri Ganganagar, IND; 2 Department of Conservative Dentistry and Endodontics, Government Dental College, Amritsar, IND

**Keywords:** computer-aided design, denture bases, polymethyl methacrylate, surface properties, wettability

## Abstract

Introduction

The surface characteristics of denture base resins, such as surface roughness, wettability, and surface hardness, are critical determinants of denture success and patient satisfaction. This in vitro study aimed to evaluate and compare the surface characteristics of three fabrication techniques: conventional heat-polymerized polymethyl methacrylate (PMMA), computer-aided design/computer-aided manufacturing (CAD/CAM)-milled PMMA, and three-dimensional (3D)-printed PMMA denture base resins. The objective of this study was to identify the most favorable material in terms of surface quality to assist clinicians in optimizing denture performance and biocompatibility.

Materials and methods

A total of 120 standardized specimens (25 × 25 × 3 mm) were prepared and divided equally into three groups (n = 40 each). Group I consisted of conventional heat-polymerized PMMA (Triplex Hot, Ivoclar Vivadent AG, Schaan, Liechtenstein). Group II consisted of CAD/CAM-milled PMMA (Ivotion base disc, Ivoclar Vivadent AG, Schaan, Liechtenstein). Group III consisted of 3D-printed PMMA (3D Accuprint Denture, D-Tech, Mumbai, India). All specimens were finished, polished, stored in distilled water, and subjected to thermocycling, followed by immersion in artificial saliva to simulate oral conditions. The surface wettability was measured using the sessile drop method and contact angle analysis. The surface roughness was evaluated using a contact profilometer (Surftest SJ-210, Mitutoyo Corporation, Kanagawa, Japan), and the surface hardness was measured using a Vickers hardness tester (Mitutoyo HM-200, Mitutoyo Corporation, Kanagawa, Japan). Data were statistically analyzed using one-way analysis of variance (ANOVA) and Tukey’s post hoc test, with significance set at p < 0.001.

Results

Significant differences were observed among the three groups (p < 0.001). The highest contact angle was recorded in the 3D-printed group (73.94 ± 2.29°), indicating greater hydrophobicity, while CAD/CAM (73.26 ± 2.37°) and conventional PMMA (68.38 ± 1.93°). The CAD/CAM specimens showed the smoothest surfaces (0.16 ± 0.014 µm), while conventional PMMA was the roughest (0.21 ± 0.019 µm). In terms of hardness, the CAD/CAM specimens exhibited the highest values, significantly outperforming the conventional and 3D-printed groups.

Conclusion

CAD/CAM-milled PMMA denture bases demonstrated superior surface smoothness and hardness, whereas 3D-printed specimens exhibited the highest hydrophobicity. Digital fabrication methods offer improved surface characteristics compared with conventional techniques, potentially enhancing the clinical performance and longevity of complete dentures.

## Introduction

Complete dentures continue to be a vital treatment modality for edentulous patients, especially in regions where access to implant-supported prostheses is limited [1]. Among the materials used for denture bases, polymethyl methacrylate (PMMA) remains the gold standard [2]. PMMA has gained widespread popularity because of its excellent aesthetics, ease of manipulation, cost-effectiveness, stability in the oral environment, and acceptable mechanical properties. Notably, approximately 95% of complete dentures are fabricated using PMMA resin [3]. Despite these advantages, conventional heat-polymerized PMMA is not without its limitations. These include polymerization shrinkage, residual monomer release, surface voids, and reduced mechanical strength, all of which may compromise the longevity, hygiene, and comfort of dentures [4].

Surface properties, such as wettability, roughness, and hardness, play critical roles in the clinical performance of denture base materials [5]. Increased surface roughness and decreased hardness can promote microbial adhesion and biofilm formation, particularly by Candida albicans, contributing to denture-induced stomatitis, a condition affecting 30-75% of denture wearers [6]. A surface roughness threshold of 0.2 μm has been suggested to minimize microbial colonization, emphasizing the need for optimal finishing and polishing procedures [7]. Moreover, poor wettability, as indicated by a high contact angle, can impair denture retention and contribute to plaque accumulation [6]. Similarly, insufficient surface hardness may predispose the prosthesis to wear, fracture, and increased porosity, further exacerbating plaque retention and soft tissue inflammation [5].

Recent advances in digital dentistry have introduced novel fabrication techniques, such as computer-aided design/computer-aided manufacturing (CAD/CAM) and three-dimensional (3D) printing, which offer promising alternatives to conventional processing [8,9]. CAD/CAM milled dentures are fabricated from pre-polymerized PMMA blocks under controlled high-pressure and high-temperature conditions [8]. This results in dentures with enhanced mechanical and surface properties, improved color stability, and significantly lower release of residual monomers. Clinical studies have also reported reduced microbial adhesion and colonization on milled denture bases, making them a viable choice for patients prone to fungal infections [10,11]. Despite these advantages, comparative data evaluating the surface properties of milled and conventionally fabricated dentures remain limited.

Additive manufacturing or 3D printing is a rapidly evolving technique in prosthodontics [9]. This method involves layer-by-layer fabrication of dentures using light-cured resins based on digital stereolithographic (STL) files. Although 3D printing allows the creation of highly detailed and customized prostheses, there is limited literature on the surface and mechanical characteristics of 3D-printed denture base materials, particularly in comparison to milled and conventional PMMA resins [12].

Given the clinical significance of surface characteristics in determining denture success and patient satisfaction, this in vitro study aimed to evaluate and compare the surface roughness, wettability, and hardness of conventional heat-polymerized PMMA, CAD/CAM-milled PMMA, and 3D-printed denture base resins. By identifying the material with the most favorable surface properties, this study sought to aid clinicians in selecting the most suitable fabrication technique, thereby enhancing the longevity, function, and biocompatibility of complete dentures.

## Materials and methods

Study design and setting

This in vitro experimental study was conducted at the Department of Prosthodontics, Maharaja Ganga Singh Dental College and Research Centre, Sri Ganganagar, Rajasthan, from May 2024 to December 2024. As this is an in vitro study, it did not involve human participants or human tissue, and ethical approval was not required.

Sample size calculation

The sample size was calculated using G*Power software (version 3.1.9.7, Heinrich-Heine-Universität Düsseldorf, Germany) for one-way analysis of variance (ANOVA). With an effect size (f) of 0.25, α = 0.05, and power = 0.95, 120 specimens were required. Accordingly, 40 specimens were allocated to each of the three study groups.

Methodology

Specimens with standardized dimensions (25 × 25 × 3 mm) were digitally designed using AutoCAD 2024 (Autodesk Inc., San Rafael, California, USA). The finalized model was exported in standard tessellation language (STL) format and milled from Plexiglass (Plazit Polygal India Pvt. Ltd., India) to serve as a template for uniform specimen fabrication across all groups. The samples were divided into three groups (n = 40 each): Group I consisted of conventional heat-activated PMMA resin (Triplex Hot, Ivoclar Vivadent AG, Schaan, Liechtenstein). Group II consisted of CAD/CAM-milled PMMA resin (Ivotion base disc, Ivoclar Vivadent AG, Schaan, Liechtenstein). Group III consisted of 3D-printed PMMA resin (3D Accuprint Denture, D-Tech, Mumbai, India).

In Group I, Plexiglass master dies were coated with petroleum jelly (Vaseline, Hindustan Unilever Ltd., Mumbai, India) and invested in a 50:50 mixture of dental plaster and Type III dental stone (Kalstone, Kalabhai Karson Pvt. Ltd., Mumbai, India). Flasking was performed in a two-part varsity flask, and a separating medium (Cold Mold Seal, Pyrex, Pyrax Polymars, Roorkee, India) was applied between the layers. Heat-cured PMMA was mixed in a 2:1 powder-to-liquid ratio and packed into a mold. Curing was performed using a short cycle of 74 °C for 2 h, followed by 100 °C for 1 h.

After polymerization, the specimens were bench-cooled, deflasked, and trimmed using a tungsten carbide acrylic bur (Komet Dental, Lemgo, Germany), followed by 400-grit silicon carbide paper (Prodec, Shanghai, China). Polishing was performed using rubber burrs (Delta Dental Products, New Delhi, India), pumice slurry (Neelkanth Healthcare Pvt. Ltd., Jodhpur, India), and rouge (Menzerna Polishing Compounds GmbH and Co. KG, Ötigheim, Germany). One surface was polished, whereas the other remained untouched. The dimensions were verified using a digital Vernier caliper (Ivoclar Vivadent AG, Schaan, Liechtenstein), and the specimens were stored in distilled water (Wellistics Life Science Pvt. Ltd., Punjab, India) for 48 h.

For Group II, the specimens were digitally designed using AutoCAD 2024 software (Autodesk Inc., San Rafael, California, USA), exported as STL files to Exocad software (Align Technology Inc., Tempe, Arizona, USA), and milled from pre-polymerized PMMA discs using a 5-axis milling machine (ARUM 5X-500, Doowon, Daejeon, South Korea). Wet milling was used to prevent overheating. The milled specimens were detached, finished, and polished following the same protocol as in Group I, and stored in distilled water (Wellistics Life Science Pvt. Ltd., Punjab, India) for 48 h.

For Group III, the 3D-printed specimens were designed using CAD software and printed using a digital light processing (DLP)-based 3D printer (Phrozen Shuffle, Phrozen Tech Co. Ltd., Hsinchu, Taiwan). PMMA resin (3D Accuprint Denture, D-Tech, Mumbai, India) was manually shaken before pouring. Printing was performed with a 50 µm layer thickness at a 45° orientation. After printing, the support structures were removed, and the specimens were ground using silicon carbide burs (Prodec, Shanghai, China) and then rinsed in 99% isopropyl alcohol (Qualiens, Thermo Fisher Scientific India Pvt. Ltd., Mumbai, India) for 3 min. The final UV curing was performed in a UV light-curing box (Phrozen Cure, Phrozen Tech Co. Ltd., Hsinchu, Taiwan) for 60 min. The finished specimens were polished similarly to the other groups and stored in distilled water (Wellistics Life Science Pvt. Ltd., Punjab, India) for 48 h. To simulate intraoral thermal fluctuations, all specimens were subjected to thermocycling using a thermocycler (SD Mechatronik Thermocycler THE-1100, Feldkirchen-Westerham, Germany). Each specimen underwent 50 thermal cycles between 5 °C and 55 °C, with a dwell time of 30 s in each bath and a transfer time of 10 s between baths. Following thermocycling, the specimens were immersed in artificial saliva (Wet Mouth, ICPA Health Products Ltd., India) for seven days to replicate oral conditions.

**Figure 1:**
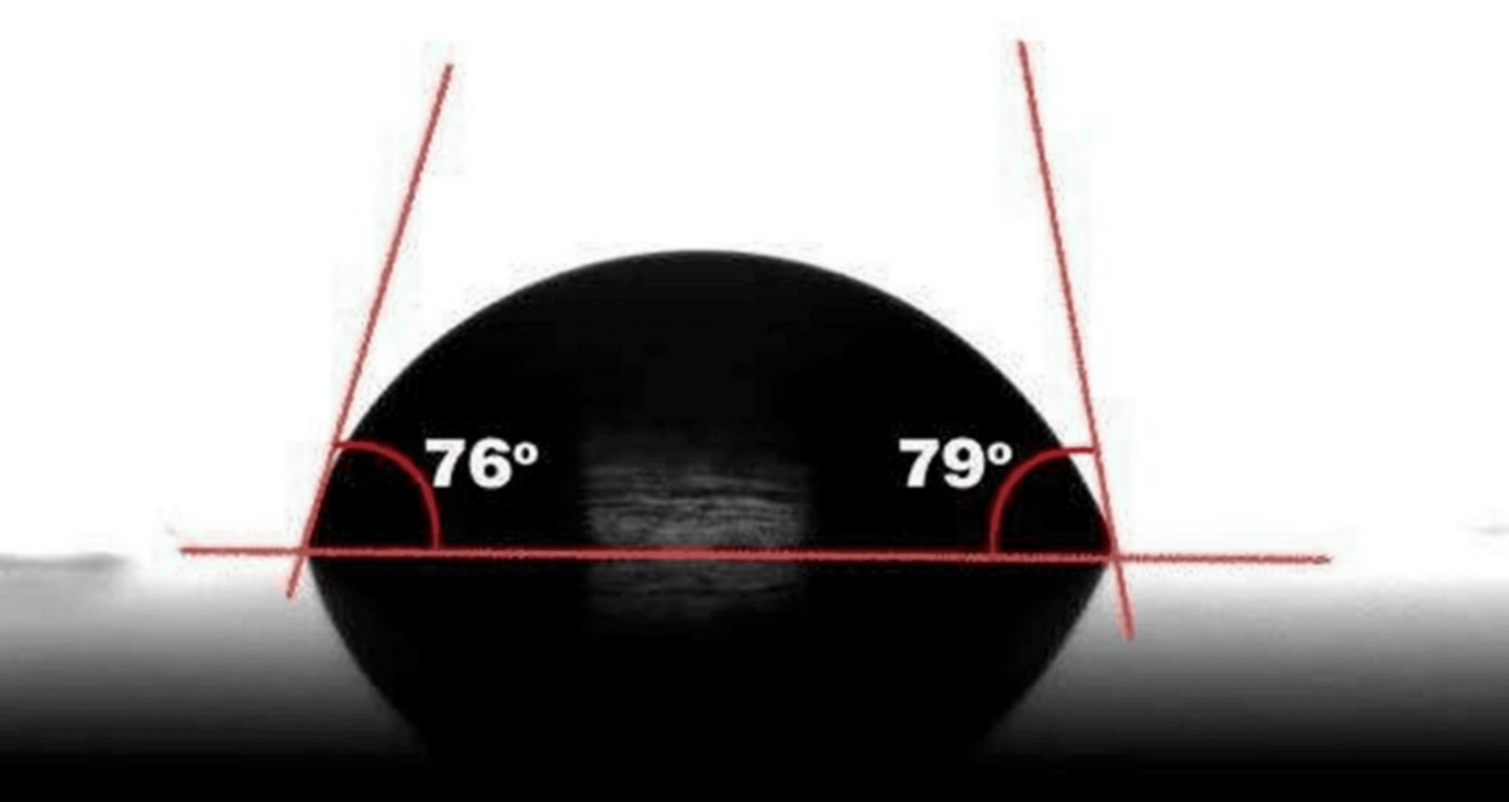
Contact angle captured using a digital single-lens reflex (DSLR) camera This figure is derived from a sample of the study.

The surface wettability was assessed using the sessile drop method. A 20 µL droplet of distilled water was dispensed onto the polished surface using a micropipette (Dragon Lab, Beijing, China). After 20 s, images were captured using a digital single-lens reflex (DSLR) camera (Canon EOS 1200D with a 100 mm macro lens, Canon Inc., Tokyo, Japan). Images were analyzed using AutoCAD 2025 (Autodesk Inc., San Rafael, California, USA) to measure the contact angles on both sides of the droplet. The average values were recorded (Figure 1).

Surface roughness was measured using a digital contact profilometer (Surftest SJ-210, Mitutoyo Corporation, Kanagawa, Japan) with 0.001 µm resolution and a tracing length of 0.8 mm. Three readings from different points on the polished surface of each specimen were recorded, and the average was calculated.

The surface hardness was assessed using a Vickers hardness tester (Mitutoyo HM-200, Mitutoyo Corporation, Kanagawa, Japan) with a 300 g load and 15-second dwell time. Three indentations were made on each specimen, and the Vickers hardness number (VHN) was calculated from the average of the diagonals.

Statistical analysis

All collected data were statistically analyzed using IBM SPSS software (version 26.0; IBM Corp., Armonk, NY, USA). Descriptive statistics, including mean and standard deviation, were calculated for all groups. One-way ANOVA was performed to evaluate differences among the groups, followed by Tukey’s post hoc test for pairwise comparisons. Statistical significance was set at p < 0.001.

## Results

One-way ANOVA revealed statistically significant differences (p < 0.001) in the mean contact angle, surface roughness, and hardness among the three groups. The 3D-printed group exhibited the highest contact angle (73.94 ± 2.29°), followed by CAD/CAM (73.26 ± 2.37°) and conventional PMMA (68.38 ± 1.93°), indicating superior hydrophobicity in the digitally fabricated groups. The surface roughness was lowest in the CAD/CAM (0.16 ± 0.014 µm), while the conventional PMMA (0.21 ± 0.019 µm) and 3D-printed (0.19 ± 0.015 µm) groups showed higher values (F = 109.06). For hardness, CAD/CAM (21.05 ± 1.28 VHN) surpassed conventional PMMA (18.92 ± 0.60 VHN) and 3D-printed (15.58 ± 0.58 VHN), suggesting material-dependent mechanical properties (Table 1).

**Table 1:** Comparison of mean contact angle (degrees), surface roughness (Ra in µm), and surface hardness (VHN) between study groups by the one-way analysis of variance (ANOVA) test *p-value < 0.05: significant, CAD/CAM: computer-aided design/computer-aided manufacturing, 3D: three-dimensional, PMMA: polymethyl methacrylate, CI: confidence interval, VHN: Vickers hardness number Data are presented in the form of mean ± standard deviation (SD).

Parameters	Groups	Mean	SD	95% CI at mean		F stat	p-value
Contact angle (degrees)	Conventional PMMA	68.38	1.93	67.76	69.00	75.45	0.001*
	CAD/CAM	73.26	2.37	72.50	74.02		
	3D-printed	73.94	2.29	73.21	74.68		
Surface roughness (Ra in µm)	Conventional PMMA	0.21	0.019	0.20	0.22	109.06	0.001*
	CAD/CAM	0.16	0.014	0.15	0.14		
	3D-printed	0.19	0.015	0.19	0.17		
Surface hardness (VHN)	Conventional PMMA	18.92	0.60	18.73	19.11	385.86	0.001*
	CAD/CAM	21.05	1.28	20.63	21.46		
	3D-printed	15.58	0.58	15.39	15.77		

Post hoc analysis revealed significant differences (p < 0.001) in all surface properties between the conventional and digital fabrication methods. The 3D-printed specimens showed significantly higher contact angles than those of the conventional (mean difference = 5.55) and CAD/CAM groups (mean difference = 0.68). The surface roughness demonstrated progressive improvement from conventional to 3D-printed to CAD/CAM, with all pairwise comparisons being statistically significant (p < 0.001). Surface hardness measurements showed that CAD/CAM specimens were significantly harder than conventional (mean difference = 2.12 VHN) and 3D-printed groups (mean difference = 5.46 VHN). Notably, while 3D printing produced more hydrophobic surfaces than CAD/CAM, this difference was not statistically significant (p = 0.357), suggesting comparable wettability between digital fabrication methods (Table 2).

**Table 2:** Pairwise comparison of study groups using post hoc Tukey’s test *p-value < 0.05: significant, CAD/CAM: computer-aided design/computer-aided manufacturing, 3D: three-dimensional, PMMA: polymethyl methacrylate, VHN: Vickers hardness number

Pairwise groups		Contact angle (degrees)		Surface roughness (µm)		Surface hardness (VHN)	
		Mean difference	p-value	Mean difference	p-value	Mean difference	p-value
Conventional PMMA	CAD/CAM	4.88	0.001*	0.054	0.001*	2.12	0.001*
Conventional PMMA	3D-printed	5.55	0.001*	0.018	0.001*	3.34	0.001*
CAD/CAM	3D-printed	0.68	0.357	0.036	0.001*	5.46	0.001*

## Discussion

This in vitro study compared the surface properties of denture base resins fabricated using three distinct techniques: CAD/CAM milling, 3D printing, and conventional compression molding with heat-cured PMMA. The surface properties analyzed were surface roughness and surface hardness, both of which are critical parameters affecting not only the aesthetics and longevity of the denture but also its clinical performance in terms of plaque accumulation, microbial colonization, and patient comfort.

The results of the current study indicate that the CAD/CAM-milled group exhibited the lowest mean surface roughness values (Ra), followed by the conventional PMMA group, while the 3D-printed group showed the highest surface roughness. This trend is consistent with several previous studies in the literature [13,14].

The superior surface smoothness in the CAD/CAM-milled group can be attributed to the industrial fabrication of pre-polymerized resin blocks under high pressure and temperature, resulting in high-density, porosity-free materials with a highly uniform microstructure [14]. Additionally, the milling process mechanically removes the prosthesis from these homogeneous blocks, reducing the introduction of surface irregularities typically observed in manual processing methods. These blocks undergo minimal dimensional changes and are less prone to internal defects, which further contributes to a smoother surface [14].

In contrast, the 3D-printed specimens displayed significantly higher roughness values, which can be explained by the layer-by-layer fabrication process inherent to additive manufacturing [11]. The stepping effect, incomplete polymerization between layers, and potential inaccuracies in resolution result in a textured surface that is not as smooth as that obtained through subtractive milling [9]. This finding is supported by a previous study that reported that 3D-printed denture bases tend to exhibit distinct layer lines and require additional post-processing to improve their surface characteristics; therefore, they showed greater surface roughness than conventional dentures [15].

Although conventional heat-cured PMMA showed moderate surface roughness in this study, it is important to consider the manual variables involved in compression molding, such as dough manipulation, flask closure pressure, and finishing/polishing techniques, all of which can affect the final surface topography [3]. Surface irregularities may also result from porosities due to residual monomer release or inadequate curing, both of which are common in conventional processing. Clinically, these findings have significant clinical implications. Denture bases with higher surface roughness can harbor more fungal species and other pathogens, thereby increasing the risk of denture stomatitis and oral infections [11]. Therefore, the use of CAD/CAM-milled denture bases may offer a clinical advantage for maintaining oral hygiene and patient satisfaction.

Surface hardness tests revealed that the CAD/CAM-milled resins again outperformed the other groups, followed by the conventional PMMA group, while the 3D-printed specimens had the lowest hardness values. This outcome aligns with the existing literature and is closely linked to the intrinsic properties of the materials and their fabrication methods [16,17].

The enhanced hardness of CAD/CAM-milled materials is primarily due to the pre-polymerized nature of the industrial blocks, which undergo polymerization under optimal and controlled conditions [17]. These blocks achieved a high degree of conversion with minimal residual monomer content, leading to improved cross-linking and densification. Consequently, these materials exhibit increased resistance to indentation and better performance against abrasive forces over time [18].

Conventional heat-cured PMMA resins, although relatively hard, are still susceptible to polymerization shrinkage, microvoids, and lower degrees of polymer conversion than CAD/CAM-milled resins [18]. These factors may compromise the mechanical properties of the material, including its hardness, even when standard laboratory techniques are followed.

In contrast, the lower surface hardness of the 3D-printed specimens can be attributed to incomplete polymerization and interlayer bonding inefficiencies [19]. The photopolymer resins used in 3D printing often depend heavily on light penetration for polymerization, and the deeper layers may not receive sufficient energy, leading to a lower cross-linking density. Inadequate post-curing procedures can further reduce the final hardness. Additionally, the presence of surface irregularities due to the printing process itself can act as a stress concentrators and weaken surface integrity [16].

From a clinical perspective, lower surface hardness can translate into faster wear, a more frequent need for polishing, and increased susceptibility to surface deterioration. This not only affects the lifespan of the prosthesis but also impacts occlusal accuracy and patient comfort. Therefore, the use of materials with superior hardness, such as CAD/CAM-milled PMMA, is beneficial for long-term prosthodontic success [18].

Moreover, Piedra-Cascón et al. emphasized the need for improved post-processing protocols in 3D printing to enhance both mechanical strength and surface integrity [20]. Their studies suggested that while 3D printing offers customization and rapid fabrication, it still lags behind in material properties unless optimized resin formulations and curing techniques are employed.

Surface wettability is a critical parameter in evaluating the performance of denture base materials, as it influences denture retention, microbial adhesion, stain resistance, and overall hygiene [11]. Wettability is typically assessed through contact angle measurements, where smaller angles signify higher wettability and hydrophilicity and larger angles indicate increased hydrophobicity. In the context of denture bases, increased wettability is generally favorable for enhancing the retention of removable dentures, as it ensures better adaptation of the denture to moist oral mucosa [21].

In the current study, the mean contact angle values were the highest for 3D-printed PMMA, followed by CAD/CAM-milled PMMA, and the lowest for conventional heat-activated PMMA. These results suggest that 3D-printed denture bases are the most hydrophobic, whereas conventional PMMA has the greatest wettability. This ranking aligns with the findings of previous studies, which similarly reported that 3D-printed materials exhibited higher contact angles and, therefore, greater hydrophobicity compared to conventionally processed PMMA [22,23]. PMMA contains carboxylate and methyl ester functional groups, which enhance its hydrophilicity and elevate its free surface energy [24].

The increased hydrophobicity of the CAD/CAM and 3D-printed PMMA may be attributed to several factors. CAD/CAM PMMA discs are pre-polymerized under high pressure and temperature, which modifies the molecular arrangement and polarity, potentially reducing the surface energy and increasing the contact angle [25]. Similarly, 3D-printed resins, owing to their layer-by-layer fabrication and photocuring processes, tend to exhibit unique surface topographies and variable degrees of polymerization, which may influence their hydrophobic behavior. Incomplete polymerization, interlayer interfaces, and surface porosity in 3D-printed specimens may all contribute to an increase in the contact angles [20].

Interestingly, while higher contact angles imply poorer wetting, Falde et al. reported that hydrophobic surfaces might be advantageous in certain clinical scenarios [26]. For instance, biofilm and bacterial detachment are often easier from hydrophobic surfaces, potentially reducing the microbial load and mitigating the risk of denture-related infections such as denture stomatitis [27]. This presents a nuanced view, while hydrophilic surfaces improve denture retention, hydrophobicity may help reduce long-term microbial colonization, highlighting a delicate balance in clinical material selection [27].

Overall, conventional PMMA showed superior wettability in this study, which may translate to improved retention in clinical applications. However, CAD/CAM and 3D-printed resins, with their higher contact angles, could offer benefits such as reduced microbial adherence and improved biocompatibility through lower residual monomer release. These findings underscore the importance of carefully selecting denture base materials based on patient-specific needs, especially in patients with poor salivary flow or susceptibility to oral infections.

Clinical implications

The findings of this study have important clinical implications for prosthodontic practice. The superior surface smoothness and hardness of the CAD/CAM-milled denture base resins suggest that they are more resistant to wear, plaque accumulation, and staining, thereby enhancing denture longevity, hygiene, and patient comfort. Although conventional heat-cured PMMA demonstrates better wettability, which is beneficial for denture retention, its relatively lower hardness and rougher surface may increase the risk of microbial colonization and maintenance issues over time. 3D-printed resins, which are convenient and cost-effective, show the least favorable surface properties, indicating the need for further material optimization or protective coatings. Therefore, selecting a denture base material should be guided by patient-specific factors, such as oral hygiene habits, mucosal conditions, and risk of infection, with CAD/CAM-milled resins offering a promising balance between mechanical durability and clinical performance.

Limitations of the study

Despite these valuable findings, this study has several limitations. As an in vitro study, it does not replicate the complex oral environment in which factors, such as salivary enzymes and pH changes, might influence surface degradation over time. Furthermore, only surface roughness and hardness were analyzed; other important parameters, such as flexural strength, impact resistance, water sorption, and microbial adherence, were not evaluated and warrant further investigation. In addition, the sample size, which was statistically valid, was relatively small. Variability in finishing and polishing procedures, particularly in the conventional group, may have influenced the results despite efforts to standardize the protocols.

Further research should include in vivo studies should be conducted to assess the clinical performance of these materials over time. Exploring the effects of mechanical wear simulations would also offer more comprehensive insights into the long-term durability. Moreover, advancements in 3D printing materials and techniques, such as hybrid resins, improved layer resolution, and enhanced curing systems, should be examined to overcome current limitations.

## Conclusions

Within the limitations of this study, it can be concluded that CAD/CAM-milled PMMA denture base resins exhibit the most favorable surface properties, including the lowest surface roughness and highest surface hardness, making them superior in terms of durability, hygiene, and clinical performance. Conventional heat-cured PMMA, while showing the best wettability, had moderate surface hardness and roughness, indicating good retention, but potentially higher susceptibility to wear and microbial adherence. 3D-printed PMMA resins, although beneficial in terms of digital customization and fabrication speed, showed the least favorable surface characteristics. Overall, CAD/CAM-milled resins appear to offer the best balance of surface properties, supporting their increased adoption in modern prosthodontic practice for the fabrication of complete dentures.
